# Combined Application of Patient-Derived Cells and Biomaterials as 3D In Vitro Tumor Models

**DOI:** 10.3390/cancers14102503

**Published:** 2022-05-19

**Authors:** Asbiel Hasbum, Ozan Karabulut, Ruben Edgar Reyes, Claudio Ricci, Alessandro Franchi, Serena Danti, Sue Anne Chew

**Affiliations:** 1 School of Medicine, University of Texas Rio Grande Valley, Edinburg, TX 78520, USA; asbiel.hasbum01@utrgv.edu; 2 Department of Health and Biomedical Sciences, University of Texas Rio Grande Valley, Brownsville, TX 78520, USA; ozan_karabulut1@baylor.edu (O.K.); ruben.reyes@edu.uag.mx (R.E.R.); 3 Department of Chemistry and Biochemistry, Baylor University, Waco, TX 76706, USA; 4 School of Medicine, Universidad Autónoma de Guadalajara, Zapopan 45129, Mexico; 5 Department of Civil and Industrial Engineering, University of Pisa, 56122 Pisa, Italy; claudio.ricci@unipi.it; 6 Department of Translational Research and New Technology in Medicine and Surgery, University of Pisa, 56126 Pisa, Italy; alessandro.franchi@unipi.it

**Keywords:** personalized therapy, scaffold, tissue engineering, primary cancer cells, experimental models, screening, 3Rs

## Abstract

**Simple Summary:**

For years, cancer has remained the second leading cause of death in U.S. and Europe even though cancer mortality has decreased, as new advances in medical treatment have made this decrease possible. Chemotherapy has remained the gold standard and “one-size-fits-all” treatment for cancer, yet this approach has lacked precision and, at times, failed. Recent studies attempt to mimic the spatial microenvironment of cancer tissue to better study chemotherapy agents by combining patient-derived cells and three-dimensional (3D) scaffold, bioprinting, spheroid, and hydrogel culturing. This commentary aims to collect and discuss recent findings concerning the combined application of biomaterials with patient-derived cancer cells to better study and test therapies in vitro, that will further personalize and facilitate the treatment of various cancers, and also address the limitation and challenges in developing these 3D models.

**Abstract:**

Although advances have been made in cancer therapy, cancer remains the second leading cause of death in the U.S. and Europe, and thus efforts to continue to study and discover better treatment methods are ongoing. Three-dimensional (3D) tumor models have shown advantages over bi-dimensional (2D) cultures in evaluating the efficacy of chemotherapy. This commentary aims to highlight the potential of combined application of biomaterials with patient-derived cancer cells as a 3D in vitro model for the study and treatment of cancer patients. Five studies were discussed which demonstrate and provided early evidence to create 3D models with accurate microenvironments that are comparable to in vivo tumors. To date, the use of patient-derived cells for a more personalized approach to healthcare in combination with biomaterials to create a 3D tumor is still relatively new and uncommon for application in clinics. Although highly promising, it is important to acknowledge the current limitations and challenges of developing these innovative in vitro models, including the need for biologists and laboratory technicians to become familiar with biomaterial scaffolds, and the effort for bioengineers to create easy-to-handle scaffolds for routine assessment.

## 1. Introduction

Although current trends show that overall cancer death rates have decreased for men, women and children, cancer remains the second leading cause of mortality in the U.S. behind cardiovascular disease and is responsible for millions of deaths worldwide [1,2]. In 2020, 1,806,590 new cases and 606,520 cancer-related deaths have been estimated. The prevalence rate for all cancers combined in the U.S. since 2018 is approximately 5% of the population. Cancer incidence rates for all ages (per 100,000 people) between 2014 and 2018 were 450.5. Between 2015 and 2019, the average mortality rate for men and women combined was 152.4 (per 100,000 men and women). Although there has been a steep decline in the death rates for melanoma and lung cancers, which can be attributed to advances in treatment such as immune checkpoint inhibitors, targeted drug therapy, and a decrease in cancer risk factors, lung cancer mortality remains the leading cause of cancer death among men and women [3]. Despite advances in novel, targeted interventions and therapeutics, chemotherapeutic drugs remain the gold standard treatment and employ a “one-size-fits-most” approach, which lack precision and result in significant variations in patient response to therapy. Recent studies have attempted to mimic the spatial microenvironment of cancer tissue to better study chemotherapy agents through various techniques such as three-dimensional (3D) scaffold, bioprinting, spheroid and hydrogel culturing 3D tumor models, which have been shown to have advantages over bidimensional (2D) cultures in evaluating the efficacy of chemotherapeutics due to their heterogeneity and simulating the tumor microenvironment [4]. Aside from evaluating the efficacy and pharmacodynamics, 3D tissue models have been used to determine toxicity and drug resistance to chemotherapeutic agents simultaneously across different cells [5]. Several research papers have been published in the last decade using cancer cell lines to build up 3D in vitro tumor models, with the promise of delivering a useful tool for personalized therapy to the patients. This commentary aims at collecting and discussing the up-to-date findings concerning the use of biomaterials with patient-derived cancer cells for a near application in the clinics.

## 2. 3D In Vitro Models for Therapy Screening: The Role of Patient-Derived Cancer Cells and Biomaterials

While considerable progress has been made in 3D bioprinting, many obstacles remain in creating tumor models that provide physiological relevance and reliable data for the development of personalized treatment. The ability to replicate tumor microenvironments and establish vasculature for appropriate oxygen and nutrient distribution to specific areas within the 3D culture are challenges that need to be addressed [4,6,7]. Despite the advantages and increased use and acceptance of 3D tumor models, they are still more expensive and time intensive than their 2D counterparts [4]. Consequently, conventional 2D models are still widely used by pharmaceutical companies for drug development even though they do not accurately represent the tumor microenvironment which limits their use for anticancer drug screening [6]. 2D cell cultures have demonstrated minimal drug resistance compared to 3D cultures which has contributed to the high failure rates in drug discovery [6]. The physiological features of tumor tissue including oxygen and nutrient delivery, gene expression, and cell proliferation are better recapitulated in 3D tissue models [4]. Factors such as immune cells, inflammatory mediators, and vasculature add complexity and significantly influence the tumor microenvironment. Thus, 3D tumor models based on patient derived cancer tissue will more closely resemble the in vivo microenvironment and have better predictive value when compared to traditional models [6].

Conventional preclinical cancer models have generally used tumor cell lines as their source of cell derivation. Immortalized cell lines have been a preference for in vitro and in vivo preclinical models because of their ease of acquisition, production, reproducibility, and proliferation rates compared to primary cells [8,9]. Cell lines are commonly used for in vivo xenograft models and for in vitro scaffolds or organoid/spheroid formation models. Santoro et al. reported differences in signaling transduction pathway by applying shear stress to 3D constructs made of Ewing sarcoma TC71 cells and electrospun scaffolds, which highlighted the importance of recapitulating not only the compositional but also the mechanical features of the tissue microenvironment in 3D bone cancer models [10]. Silk scaffolded 786-O cell models developed by Abbott et al. showed increased lipid drop development, a significant upregulation of genes that signal cytokines and immune checkpoint inhibition therapy markers as well as downregulation of the genes CXCLS, ACACA, FASN, and CD10 comparatively between 2D and 3D scaffolded 786-O cells [11]. Persson et al. reported distinct differences of proteins between 2D and 3D tumor model as well as larger variations and more diversity between the secretome of all 3D cultures [12]. Additionally, they found correlation of the proteins secreted and clinical parameters of the original breast cancer [12]. Sun et al. utilized purchased HepG2 cells to 3D print an effective tumor model and compare it to a conventional 2D model using the same HepG2 cells. The 3D model showed significantly higher levels of mRNA related to liver function, higher expression of liver-associated proteins, a differing gene expression profile, and large differences in drug resistance [13]. 

Despite their upsides, immortalized cell lines have a handful of drawbacks that significantly affect their overall effectiveness as tumor modeling agents. Their biggest drawback occurs in their production; because cells derived from immortalized cell lines must have uncontrolled tumor-like growth in ex vivo conditions, they may suffer from altered genetic material, differing biological or tumorigenic properties compared to primary cells. Pan et al. discovered proteomic differences of the hepatoma cell line Hepa1-6 compared to primary hepatocytes [9]. Other tumor cell lines, like those of ovarian, breast, and neck cancer, also showed higher rates of mutations in comparison to their primary cell counterparts [14,15,16]. Because of the inevitable differences of cell lines to primary cells, in addition to the inherent individuality of every tumor, using cell lines as a preclinical tumor modeling tool may not be accurate. Alternatively, using patient derived cells as tumor models may guarantee increased similarity in biological and tumorigenic properties.

Patient-derived cells for use in 3D models offer a unique and relatively new approach to tumors. Unlike tumor cell lines that have garnered phenotypic and functional changes throughout their use, patient-derived cell models allow for the retention of most biochemical and physiological features from the in vivo tissue [17,18,19]. Furthermore, various studies have shown the possibility of using these patient-derived cells to determine drug combinations and resistance and identify the most effective treatment [19,20]. Patient-derived cells open the door to more targeted treatment, however, still carry some challenges due to the limited availability of these cells and difficulty in proliferating them as a result of tumor cell senescence [21,22]. The comparison of cell lines and patient-derived cells is summarized in Table 1.

Advancements in tissue engineering have slowly allowed biomaterials to play a bigger role in creating these 3D cancer models than using only cells (e.g., spheroids). Not only do biomaterials allow for a more realistic 3D structure, but they offer more realistic cell-to-cell interactions and microenvironments as opposed to 2D models [23,24,25]. Additionally, Rao et al. demonstrated that biomaterials can be used to monitor tumor progression and metastases [26]. Biomaterials can be natural materials, such as alginate, hyaluronic acid, gelatin, which tend to be highly biocompatible and can be degraded enzymatically by the body; however, they are often associated with immunogenicity or homogeneity [27]. Alginate is a naturally occurring anionic polysaccharide that can easily gel by ionic crosslinking using divalent metal ions, such as calcium. The ability to gel allows cancer cells to be encapsulated in alginate microparticles or scaffolds. DelNero et al. utilized alginate-based 3D scaffolds that can control oxygen concentration, resulting in homogeneous oxygen levels in the scaffold which permitted them to better study tumor hypoxia and angiogenesis [28]. Chitosan is a natural cationic polysaccharide, which is obtained from chitin, present in arthropod exoskeletons and some mushrooms, after deacetylation. It can be used to produce films, fibers or porous scaffolds and thus, is a very versatile biomaterial [29,30]. Dhiman et al. employed chitosan scaffolds for the culture of breast cancer cell lines and determined that chitosan polymer with high degree of deacetylation favored adsorption and cell growth [31]. Collagen is an important protein in tissues for physical support [32]. Duarte Campos et al. applied collagen as bioprintable bioinks and demonstrated that, since the printed bioink was stable enough, cells seeded in the bioprinted models maintained their ability to proliferate. They suggest that this biomaterial may be promising to be used with patient-derived primary tumor cells for precision medicine therapy [33]. Gelatin is another natural polymer that is widely used as a biomaterial. It is the denatured form of collagen, and unlike collagen which has low water solubility, it is a water-soluble biomaterial. Nii et al. utilized gelatin to fabricate microparticles as a 3D cell culture system combined with drug delivery, as a cancer invasion model [27]. Hyaluronic acid is a key ECM component and thus, is a suitable biomaterial for 3D cell culture. Engel et al. fabricated a multi-layered hyaluronic acid hydrogel to coculture cancer and stromal cells and demonstrated that it can improve drug screening predictability compared to 2D cultures [34].

Among synthetic polymers, aliphatic polyesters are another class of biomaterials that is usually biodegraded through hydrolysis and their characteristics can be controlled easily [27]. One of the synthetic polymer that is widely used for 3D biomaterial models is poly(lactic-co-glycolic acid) (PLGA), whose degradation rate can be altered by controlling the lactic and glycolic acid ratio, the polymer molecular weight, as well as its end caps. Luo et al. demonstrated that PLGA electrospun scaffold incorporating hydroxyapatite (HA), which is often used for bone regeneration, can better support cancer cells compared to PLGA scaffold alone as the HA induce cell growth, DNA synthesis and cell division [35]. A biodegradable polymer widely used as a biomaterial is polycaprolactone (PCL), which is also a polyester like PLGA and thus, degrades by hydrolysis. Chen et al. utilized PCL to 3D print scaffolds and co-cultured colorectal cancer cells, cancer-associated fibroblasts and tumor-associated endothelial cells on them to develop a 3D model of tumor tissue for colorectal cancer [36].

Natural polymers, due to their water solubility, usually result in hydrogels, which are common types of biomaterials for 3D cancer models due to their ease of encapsulating the cells of interest. Synthetic polymers, which are usually more hydrophobic in nature are thermoplastic and soluble in non-water solvents. Therefore, they often result in scaffolds fabricated either a sponge or fiber form, obtained via emulsion, compression molding, 3D printing or electrospinning, among other methods. Such prefabricated structures can offer microenvironments for 3D cancer models, in which geometrical features, porosity, mechanical properties and roughness can be all tuned. Just like hydrogels, they provide the vital cell–cell and cell-ECM interactions that would mimic real tumors. Additionally, these scaffolds account for controlled mechanical properties, thus can be stiffer and more stable and can better withstand shear stress, as opposed to hydrogels [37].

## 3. Combined Application of Biomaterials and Patient-Derived Cancer Cells

**Figure 1:**
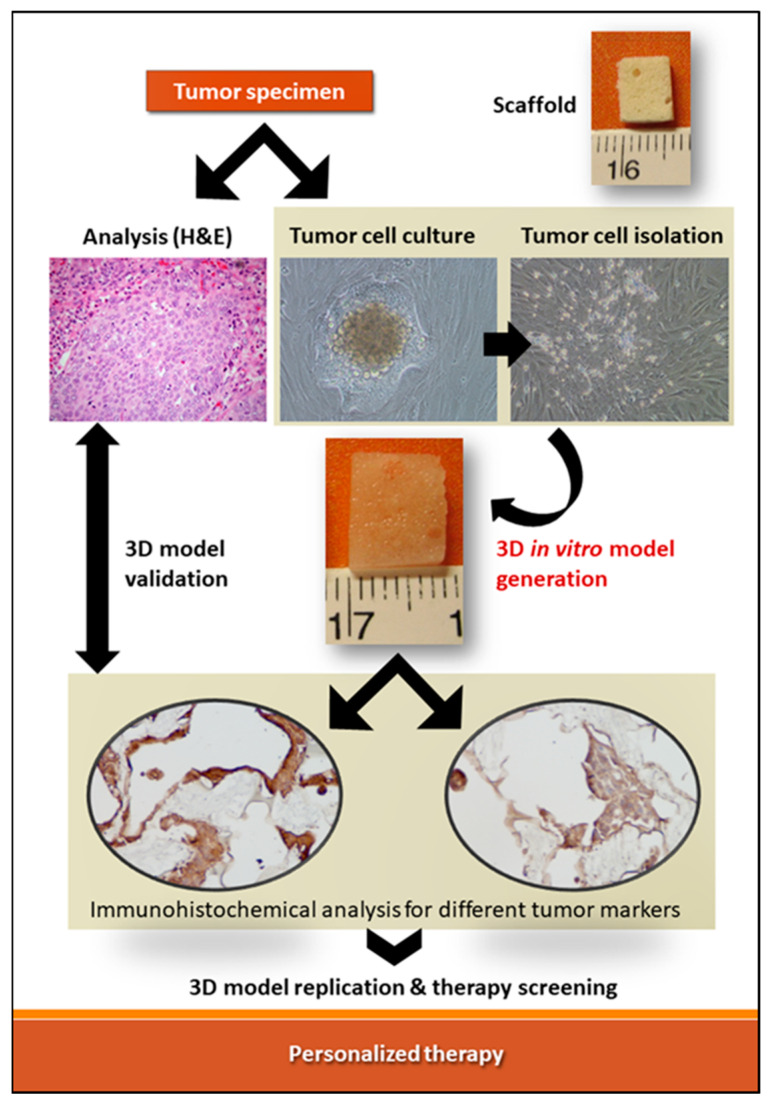
Schematic depicting the combined application of patient’s larynx tumor-derived cells (TCCR3) and polyvinyl alcohol (PVA) spongy scaffold to create 3D in vitro tumor models. Immunohistochemical analysis shows immunopositivity (in brown) for Integrin α5 (on the left) and Smad4 (on the right). All figures in the schematic are original unpublished material of the authors.

Personalized healthcare is a much more effective form of treatment for very complex diseases that have been, for the longest time, receiving broad and comparatively generic treatments. Tumors and their complete environment in a body are incredibly complex and individualized and the utilization of patient-derived cells with the combination of biomaterials to create a 3D model may be a promising method to address this challenge. Cell lines are most often used to create a 3D in vitro model, while patient derived cells are most often used as patient derived xenographs (PDX) models in vivo models. Comparatively, very few studies use both patient derived cells in tandem with biomaterials to create an in vitro 3D models of tumors. The flow chart explaining the process to build those models for personalized therapy is shown in Figure 1.

There are many applications of biomaterials as 3D in vitro tumor models; however, they are usually coupled with cell lines instead of patient-derived cells. For example, Sun et al. utilized bioprinted scaffolds seeded with hepatocellular carcinoma HepG2 cell line [13] and Abbot et al. utilized silk scaffold seeded with renal cell carcinoma 786-O cell line [11]. Patient-derived tissues have also been utilized as 3D tumor models by creating patient-derived scaffolds (PDS) that are obtained via decellularization of surgically resected tumors and then used as a substrate for cell line culture [38]. Parkinson et al. cultured colon cancer cell line HT29 in PDSs [38]. They demonstrated that the PDSs can result in induced transcriptomic and proteomic responses that align with patient-specific clinical disease information and thus, can be a potential tumor model for predicting the effectiveness of cancer therapies. Pearson et al. recellularized PDSs with breast cancer cell lines, MCF7 and MDA-MB-231 [12], whereas Gustafsson et al. used PDS to culture breast cancer cell lines MCF7 and T47D [39], as in vitro 3D tumor models. Besides PDX and PDS, fresh tumor samples, employed directly without separating the cells by enzymatic or mechanical digestion from the tumor specimens, have also been applied as ex vivo 3D tumor models [40]. These 3D tumor models conserved the original tissue architecture and cell heterogeneity better than other 3D models where the cells are removed from the original tumor [41,42]; however, they cannot be used for longer cultures and passages (i.e., usually can be culture for a week) [40]. Therefore, such tumor explants although able to mimic the TME very well, may not be as cost effective and easily accessible models.

To the best of our knowledge, there are only a few studies reporting the combination of both patient-derived cells and biomaterials as 3D in vitro models, which are summarized in Table 2. Below, we discuss each of these applications.

### 3.1. Polymeric Scaffolds and Primary Pancreatic Ductal Adenocarcinoma Cells

**Figure 2:**
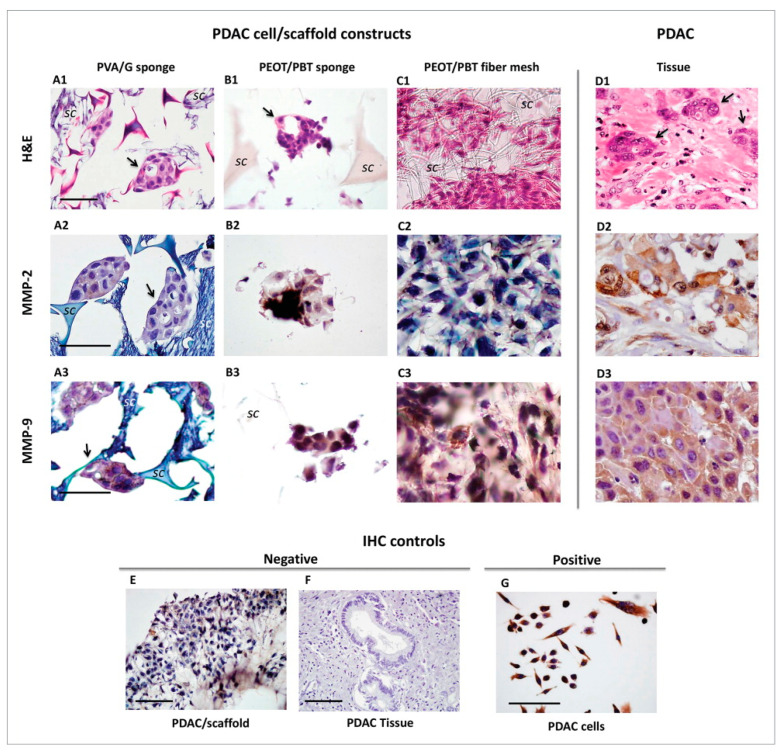
Histological micrographs of (**A**–**C**) pancreatic ductal adenocarcinoma (PDAC) cell/scaffold constructs, and (**D**) tumor tissue: (**A**) PVA/G spongy scaffold prepared via emulsion and freeze-drying, (**B**) PEOT/PBT spongy scaffold prepared via compression molding and salt leaching, and (**C**) PEOT/PBT fiber mesh prepared via electrospinning. (**A1**–**D1**) Hematoxylin and eosin staining, and (**A2**–**D2**, **A3**–**D3**) immunohistochemistry for metalloproteinases (MMPs) MMP-2 and MMP-9. Arrows indicate some organized clusters of cells with duct formation; “sc” indicates the scaffold material. (**E**–**G**) Controls of immunohistochemical reactions. Scale bar is 50 µm. (Reprinted from Taylor & Francis, Ricci et al., Biomatter, 2014 (Ricci, 2014 [43]).

In their study, Ricci et al. isolated a pancreatic ductal adenocarcinoma (PDAC) cells from an explant of a patient, created different scaffold architectures, and used the scaffold as well as the PDAC cells in conjunction to explore biomaterial-based 3D tumor models [43]. Three different polymeric scaffolds architectures were used in the creation of the 3D in vitro tumor models of the patient-derived PDAC [43]. The tumor samples were obtained from surgical procedures and cleaned thoroughly before use. The study used polyvinyl alcohol (PVA)/gelatin (G) at 80/20 (*w*/*w*)% and a poly(ethylene oxide terephthalate)/poly(butylene terephthalate) (PEOT/PBT) copolymer as their structure materials [43]. PDAC cells preferred to aggregate in sponge-like material rather than nanofiber structures and preferred the PVA/G sponge compared to PEOT/PBT sponge and PEOT/PBT fiber mesh [43]. Matrix metalloproteinases (MMPs) are enzymes that are important in protein degradation and have been directly correlated with cancer development and invasion [48]. MMP-2 and MMP-9 in particularly, because of their relationship with the tumor suppressor Smad4, which is downregulated in PDAC progression [49,50], are important factors to study PDAC. As shown in section A2 in Figure 2, PDAC cells in PVA/G sponges had higher levels of active MMP-2 production and protein synthesis [43]. Results in section A3–D3 of Figure 2 demonstrate that MMP-9 expression was strongly positive in all three cell/scaffold constructs. Nonetheless, PDAC cell MMP-2 and MMP-9 expression in the PEOT/PBT copolymer structure is still reduced in the production of active MMP-9 [43].

In conclusion, this study showed the possibility of using various biomaterial scaffolds with patient-derived cells to find a compatible pair that could work to model real pancreatic tumors. Among them, spongy scaffolds, like those obtained via PVA/G emulsion and freeze-drying, were the most suitable. They showed volume swelling ratio higher than 200% and were mechanically soft, with material stiffness increasing with G content ≥20%, due to enhanced sites of G crosslinked by glutaraldehyde [51].

The comparative analyses demonstrated that PVA/G 70/30 and PVA/G 80/20 (*w*/*w*)% were similar in terms of morphology, swelling behavior, water stability, physico-chemical and viscoelastic mechanical properties, with an apparent compressive modulus of about 7 kPa at strain rates of 0.005 s^−1^ [51]. In dry conditions, PVA/G 80/20 (*w*/*w*)% sponges showed high volume porosity (i.e., 84.43%) and pore interconnectivity (97.44%), the latter under pore-pore openings ≥51.2 µm [52]. It is possible that this scaffold could mimic the morphological and mechanical features of the pancreas, whose stiffness increased from 7.72 to 10.97 kPa under shear wave velocity measurements, from normal to fibrotic organs [53].

The easy procedure leading to the fabrication of such scaffolds and their usefulness in pancreatic cancer in vitro modeling was described by Ricci et al. [54]. Among other interesting characteristics, such as durability (i.e., non-biodegradability, thus suitability even for long term cell cultures, since mechanical and pore properties remain consistent over time), PVA/G sponges were fully processable via routine histology processing, which made them interesting scaffolds for in-hospital research. In fact, it is possible to directly compare the generated 3D model with the patient’s tumor under histology; therefore, the morphological features of the cells and the newly formed in vitro tumors can support the suitability of the 3D model for a possible therapeutic screening. s [51,54]. This same principle could be exploited to model other types of cancers throughout their various phases and screen for drug susceptibility.

### 3.2. Bioprinting of Patient-Derived Intrahepatic Cholangiocarcinoma

In this study, Mao et al. used a bioprinting process to create a 3D model of intrahepatic cholangiocarcinoma. Bioprinting offers uniqueness to creating 3D tumor models; while other modeling procedures have random arrangements and densities of molecules, cells, and biomaterials, bioprinting offers control over their density, arrangement, and structural design [44]. This study also used patient-derived tumor specimens acquired through a resection surgery on a single man diagnosed with intrahepatic cholangiocarcinoma and a composite hydrogel system containing gelatin-alginate-Matrigel to print their tumor model [44]. The 3D printed culture exhibited a more uniform distribution of cell clusters, as well as a faster aggregation process, in comparison to the sandwich culture [44]. Tumor markers CA19-9 and CEA, cancer stem cell markers CD133 and EpCAM, relative gene expression, liver function markers, as well as pathological markers of ICC cells were significantly higher in the 3D tumor model microenvironment [44]. Additionally, the MMP and fibrosis makers of the 3D model were significantly higher and showed much better drug resistance than the sandwich model [44]. This drug resistance opens the possibility of using these models as personalized therapy due to displaying stem-like properties. Overall, this study demonstrated the possibility of retaining cell viability while bioprinting, the vital up-regulation of tumorigenic phenotypes in 3D models when compared to 2D models, and the potential of using these models to study drug resistance for a more personalized treatment.

### 3.3. Porous Scaffold Composed from Bombyxmoricocoons Silk Was Infused with Liquid ECM Gel for Pediatric Brain Tumors

**Figure 3:**
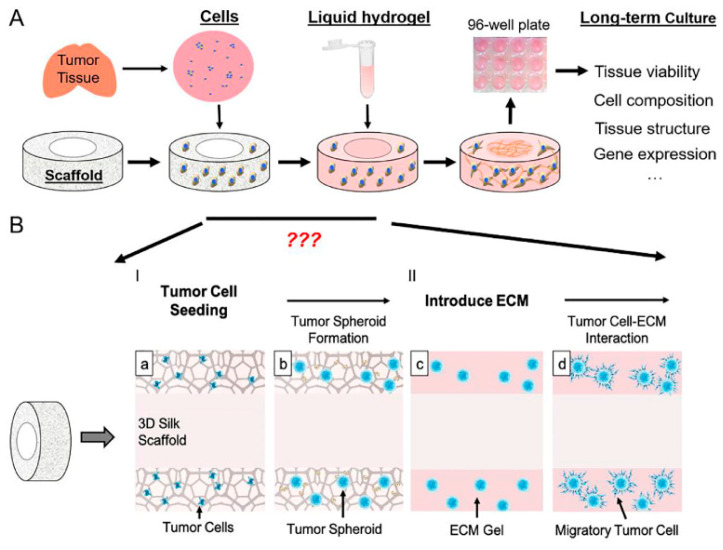
Schematics of the 3D modeling process: (**A**) Schematics of the 3D brain tissue engineering process. (**B**) To adapt the process for brain tumor model, questions regarding media conditions, ECM and timing for the change of culture conditions need to be addressed. Dissociated tumor cells are seeded onto a donut-shaped 3D silk-based porous scaffold, from which tumor spheroid develops. ECM gels are introduced to the scaffold filling the pores and the center-hole (CH) region, providing a permissive environment for the migrating tumor cells and cell–cell interaction. (**a**) Tumore cell seeding, (**b**) tumor spheroid formation, (**c**) introduce ECM, (**d**) tumor cell-ECM interaction. (Reprinted from Elsevier, Tang-Schomer et al., Translational Oncology, 2022 [45]).

Porous scaffold composed from Bombyxmoricocoons silk was infused with liquid ECM gel (Figure 3) and employed for a variety of pediatric brain tumors, namely, 11 pediatric tumor cases, consisting of three medulloblastoma (MB), three ependymoma (EPN), one glioblastoma (GBM), and four juvenile pilocytic astrocytoma (Ast) patients [45]. Tang-Schomer et al. found that the 3D scaffold alone supported cell heterogeneity and had the ability to form tumor type-dependent spheroids, which were not possible in 2D or gel-only control cultures. They concluded that the 3D scaffold silk-based structure had a vital role in supporting tumor spheroids, giving structural stability to gels, and maintaining tumor stem cells in 3D.

### 3.4. Co-Assembly of Peptide Amphiphiles (PAs) with Custom ECM Components (PA-ECM) with Pancreatic Ductal Adenocarcinoma (PDAC)

Osuna de la Pena et al. cocultured patient-derived cells obtained from PDAC with a 3D co-assembly of peptide amphiphiles (PAs) with custom ECM components (PA-ECM) for ex vivo tissue modeling with increased adaptability [46]. This model of PDAC was able to sustain patient-specific transcriptional profiles and demonstrated high cancer stem cell functionality. These peptides provided a reductionist approach to bioengineering complicated microenvironments by regulating nanoscale geometries and epitope presentation to selectively signal cells.

### 3.5. Patient-Derived Malignant Hematopoietic Stem Cells in HA Scaffold Developed with a Bioreactor System for Acute Myeloid Leukemia and Myeloproliferative Neoplasms

Andrés García-García et al. demonstrated how cellular niches may be built in a 3D hydroxyapatite scaffold and perfusion flow-based bioreactor system and used to maintain, expand, and regulate the phenotypic and functional properties of patient-derived human malignant hematopoietic stem and progenitor cells (HSPCs) ex vivo [47]. The fully humanized model was used to study human leukemogenesis in the presence of tailored niche components (e.g., osteoblastic vs. stromal-vascular elements) and to assess chemotherapeutic responsiveness. Human osteoblastic bone marrow niches were produced by culturing mesenchymal stromal cells in the scaffolds under perfusion flow in a bioreactor system. They demonstrated that the 3D model provided an environment that could sustain CD34^+^ cells from acute myeloid leukemia (AML) and myeloproliferative neoplasm (MPN) patients for up to 3 weeks.

## 4. Conclusions

The use of patient-derived cells for a more personalized approach to healthcare and biomaterials to create 3D tumor are relatively new and uncommon. Although highly promising due to their more accurate cellular environments that mimics that of the patient, it is important to acknowledge the current limitations of these innovative in vitro models. From having to mimic the molecular biology, physiology and genetic makeup of these cancer cells and ultimately reproduce the heterogenous nature of cancer cells, these models face multiple obstacles. A key challenge is developing a way to create a network of vasculatures in tumors. Without these capillary networks, the 3D tumor growth would be difficult and possibly impossible to occur. Nonetheless, studies have shown that if these bioprinted models are able to account for cellular and molecular factors, it is possible to transform stem cells into endothelial cells, thus promoting angiogenesis [55,56]. Moreover, recreating these complex microenvironments has deemed difficult as various studies have shown a multitude of cancers interacting with surrounding stromal cells and environmental factors to further their growth. This sets up a challenge as it is not as simple as combining these cells and factors with each other and expecting the same system to exist [57,58,59]. Cell viability has played a factor in the creation of these 3D models as the shear forces of bioprinting often damages the cells ultimately deeming them unusable. Current technology has made it possible for significant progress to occur; nonetheless, it is still very limited due to the expensive and time-consuming process to create these personalized and accurate 3D tumor microenvironments.

As technology and knowledge about how these cancers rapidly progresses, current conventional cancer models still carry several limitations. In future studies, it will be vital to minimize the cost and focus on the development and fabrication of less time-consuming models, while accounting the importance of every cellular and molecular factors when using patient-derived cells and designing these 3D tumor constructs. Furthermore, keeping cell viability and replicating the complex environment of tumors, such as vascular network and surrounding stromal cell to cell interactions, are areas of research that ongoing and future studies are and should focus on. A recent study by Contessi Negrini et al. has suggested the use of human mesenchymal stromal cells to pregenerate bone ECM on a 3D printed polyurethane scaffold, to be used, after cell lysis, as a bioactive and biomimetic environment for osteosarcoma cell growth [60], which demonstrates the possibility of integrating a synthetic biomaterial with biomolecules produced by patient-derived healthy cells to replicate the some complexity of the TME. Overall, the incredible effectiveness of biomaterial-based scaffolding and 3D printing has shown remarkable promise, hence, allowing a more personalized healthcare approach; however, much work is needed to continue to progress in the development of these in vitro systems.

## Figures and Tables

**Table 1:** Comparison of cell lines vs. patient-derived cells.

	Cell Lines	Patient-Derived Cells
**Accessibility**	Easily accessible	Difficult to access, limited availability of cells/tissues
**Cost**	Low cost to obtain and culture	Increased cost to obtain and culture
**Proliferation**	Proliferates rapidly and indefinitely	More difficult to proliferate due to tumor cell senescence, limited amount of pasages
**Ease in culturing**	Robust and easy to work with and maintain	More fragile and difficult to work with and maintain
**Reproducibility**	Pure population of cells therefore reproducible data can be obtained	Heterogenous population of cells therefore data can differ between cell populations
**Ability to mimic TME and clinical response**	Lack of complexity to mimic tumor environment and clinical response	Can better mimic the TME and clinical response
**Ethical issues and research compliance**	No ethical concerns and no need for institutional review board approval to obtain and use	Need to obtain institutional review board approval to obtain and use

Tumor microenvironment (TME).

**Table 2:** 3D in vitro models that utilize patient-derived cells and biomaterials.

Cancer Type	Type of Cells	Biomaterial/Scaffold	Main Outcome	Ref.
Pancreatic ductal adenocarcinoma (PDAC)	Cells obtained from PDAC pieces/explants	(PVA/G) blend sponges; (PEOT/PBT) copolymer compression molded scaffolds and electrospun fibers meshes.	PDAC cells demonstrated various behaviors when exposed to different scaffold types. Sponge-like pores allowed for cellular clustering resembling the native cancer morphostructure. In PVA/G sponges the active MMP-2 enzyme was the highest.	[43]
Intrahepatic cholangiocarcinoma (ICC)	Cells obtained from a male patient diagnosed with ICC	Gelatin-alginate-Matrigel™ hydrogel bioink	Printed ICC cells showed colony forming capacity, high survival rate, active proliferation, invasive and metastatic phenotype and other characteristics of ICC cells, e.g., expression levels of tumor markers and cancer stem cell markers.	[44]
Pediatric brain tumors	Cells obtained from 11 pediatric tumor cases, consisting of three medulloblastoma (MB) patients, three ependymoma (EPN) patients, one glioblastoma (GBM) patient, and four juvenile pilocytic astrocytoma (Ast) patients.	Bombyxmoricocoons silk porous scaffold infused with liquid ECM gel	The 3D scaffold silk base structure had a vital role in supporting tumor spheroids, giving structural stability to gels, and maintaining tumor stem cells in 3D.	[45]
PDAC	Cells obtained from PDAC patient	Co-assembly of PAs with custom ECM components (PA-ECM)	This model of PDAC was able to sustain patient-specific transcriptional profiles and demonstrated high cancer stem cell functionality.	[46]
Acute myeloid leukemia (AML) and myeloproliferative neoplasms (MPN)	Cells obtained from malignant human malignant hematopoietic stem and progenitor cells (HSPCs)	HA scaffold with perfusion bioreactor	The 3D model provided an environment that could sustain CD34^+^ cells from acute myeloid leukemia (AML) and myeloproliferative neoplasm patients for up to 3 weeks.	[47]

Abbreviated as follows: Poly(vinyl alcohol)/gelatin (PVA/G); poly(ethylene oxide terephthalate)/poly(butylene terephthalate) (PEOT/PBT); extracellular matrix (ECM); peptide amphiphiles (PAs); Hydroxyapatite (HA).

## Data Availability

Not applicable.
